# Effects of Photoperiod on the Developmental Duration and Reproduction of *Sclerodermus sichuanensis*

**DOI:** 10.3390/insects16070701

**Published:** 2025-07-08

**Authors:** Kui Kang, Lina Wang, Zhongjiu Xiao, Shaobo Wang, Ke Wei, Xiaoyi Wang, Yanlong Zhang, Yanlong Tang

**Affiliations:** 1 College of Biological and Agricultural Science and Technology, Zunyi Normal University, Zunyi 563002, China; hzausgkk@163.com (K.K.); wanglina828@163.com (L.W.); xzj1989099@163.com (Z.X.); 18848966357@163.com (S.W.); 2 Key Laboratory of Forest Protection of National Forestry and Grassland Administration, Ecology and Nature Conservation Institute, Chinese Academy of Forestry, Beijing 100091, China; weike@caf.ac.cn (K.W.); xywang@caf.ac.cn (X.W.); zhangyanlong1981@163.com (Y.Z.)

**Keywords:** *Sclerodermus sichuanensis*, photoperiod, development, reproduction

## Abstract

*Sclerodermus sichuanensis*, a parasitic wasp, plays a significant role in wood-boring forest pest control in China. Research has shown that the photoperiod significantly affects the development and reproduction of parasitic wasps. However, the effects of the photoperiod on *S. sichuanensis* have not yet been reported. This study investigates the impact of different photoperiods on the developmental duration and reproduction of *S. sichuanensis*. The wasps were reared under four photoperiod conditions: 0L:24D, 8L:16D, 16L:8D, and 24L:0D. The results show that increased light duration shortened the egg, larval, and pupal stages, with the total developmental period decreasing by up to 17 days. The number of offspring was highest in the 8L:16D and 16L:8D treatments, while constant light (24L:0D) led to a significant decrease in offspring numbers. The parasitism rate and pre-oviposition period were also affected by light exposure, with the longest pre-oviposition period observed in complete darkness. These findings indicate that the photoperiod plays a crucial role in regulating the development and reproductive efficiency of *S. sichuanensis*, suggesting that appropriate light conditions could enhance the efficiency of its use in pest control.

## Simple Summary

This study examines how different light conditions influence the development and reproduction of the parasitic wasp *Sclerodermus sichuanensis*, which is used in pest control. It was found that longer periods of light reduce the time it takes for the wasp to develop from an egg to an adult, while also increasing the number of offspring. However, constant light reduced the number of offspring. The pre-oviposition period, the time before the female wasp starts laying eggs, was longest in complete darkness. These results highlight the importance of managing light conditions when rearing *S. sichuanensis* for pest control to optimize its effectiveness.

## 1. Introduction

*Sclerodermus sichuanensis* Xiao 1995, a parasitic wasp belonging to the family *Bethylidae* in the order Hymenoptera, was first discovered in Sichuan, China, in 1994. It is a multi-host parasitic species that primarily targets *Semanotus sionanster* Gressitt, a wood-boring pest [1,2]. Subsequent studies have revealed that *S. sichuanensis* possesses strong host-searching abilities, a broad host range, and high reproductive potential, making it a promising biological control agent. It is particularly effective against medium- and small-sized wood-boring pests, such as *Callidium villosulum* Fairmaire, *Semanotus bifasciatus* Motschulsky, and *Clytus validus* Fairmaire [3]. For larger pests, such as *Monochamus alternatus* Hope [4], *Aromia bungii* Faldermann [5], and *Batocera horsfieldi* Hope [6], *S. sichuanensis* can effectively parasitize early instar larvae, but its ability diminishes as host larvae grow larger. This species has become one of the most widely used natural enemies in China, and understanding the factors influencing its development and reproduction is crucial for optimizing its rearing and utilization [7].

Parasitic wasp development and reproduction are influenced by numerous factors, including biotic factors, such as host quality and microbial interactions [8,9], as well as abiotic factors, such as temperature and photoperiod [10,11,12,13]. Photoperiod, in particular, plays a critical role not only in diapause induction and termination [11,14] but also in influencing development and reproduction [15,16]. For example, the developmental duration of *Coccophagus japonicus* Compere is shortest under a 12L:12D photoperiod, which also maximizes offspring production [16]. Excessive or insufficient light negatively affects the growth and development of *Aphidius colemani* Viereck [15]. A 15L:9D photoperiod enhances the reproductive capacity of *Trichogramma dendrolimi* Matsumura populations [17]. The female-to-male ratio of *Anisopteromalus calandrae* Howard is the lowest under constant light conditions [18]. Photoperiods also affect parasitism rates. The parasitic ability of *Trichogramma japonicum* Ashmead is significantly higher under an 8L:16D photoperiod than under 12L:12D or 16L:8D [19]. A 12L:12D photoperiod maximizes parasitism rates in *Bracon hebetor* Say [20].

Photoperiod information is detected by specific photoreceptors and transmitted to a photoperiodic clock, which measures the length of the light or dark phase to classify the photoperiod as a long day (LD) or short day (SD) [21]. LD or SD information is then transmitted to regulatory systems that induce diapause or reduced growth rates or, conversely, non-diapause or accelerated growth responses [22,23,24]. Under natural conditions, the developmental rate of insect larvae is regulated by temperature and photoperiod [25]. These two factors influence larval development via different pathways. Research by Miki et al. on the cricket *Modicogryllus siamensis* Chopard showed that temperature affects larval development through the insulin/TOR signaling pathway, whereas photoperiod influences larval development through juvenile hormone (JH) [25]. The action of JH is mediated by the JH-inducible gene *Krüppel homolog* 1 (Kr-h1). The final step in JH production, which converts inactive JH precursors to active JH, is controlled by JH acid O-methyltransferase (JHAMT) [26,27]. The photoperiod also significantly influenced the pupal developmental duration of *S. sichuanensis*. Longer light duration led to shorter pupal periods. Under constant light, the pupal duration was shortened by 10 days compared with complete darkness, averaging just 13.9 days. Studies have demonstrated that under short photoperiods, the pupal developmental duration of the non-diapause *Helicoverpa armigera* Hübner is longer than that under long photoperiods [28]. Similarly, the pupal duration of *Agrotis ypsilon* Rottemberg decreases with increasing light exposure [29]. Research indicates that lipid metabolism during fat body dissociation affects the hemolymph lipid content, thereby influencing pupal development [30].

This study investigated the developmental duration and offspring quantity of *S. sichuanensis* under different photoperiods to clarify the response of the parasitoid to the photoperiod. These findings not only contribute to a deeper understanding of its biological characteristics but also provide a theoretical foundation for further exploration of the impact of the molecular circadian clock on *S. sichuanensis*. These results have significant implications for breeding and other practical applications.

## 2. Materials and Methods

### 2.1. Insects

*Sclerodermus sichuanensis* Xiao was collected in 2020 from Luzhou (29.141 N, 105.392 E), Sichuan Province, China. The substitute host, *Thyestilla gebleri* Faldermann, was collected from the roots of infested *Abutilon theophrasti* Medicus from Dagang District (38°56′ N, 117°29′ E), Tianjin City, China. The parasitoid colony was established in the laboratory using *T. gebleri* larvae as the host under the standard conditions of temperature 25 ± 1 °C, 60–70% relative humidity, and a 12 h light:12 h dark regime. These populations have been maintained in laboratory cultures for 20 generations.

### 2.2. Inoculation of Host Larvae with Parasitoids

In this experiment, 140 *T. gebleri* Faldermann larvae (weight, 200.0–240.0 mg) were selected for rearing the parasitoids and were randomly and equally divided into four groups, each group including 35 repetitions. For each replicate, we randomly selected a healthy, one-week-old, mated wingless female parasitoid. Each parasitoid was placed in a small glass vial (diameter: 1 cm; length: 5 cm) containing one *T. gebleri* larva, and each vial was closed with a cotton plug. Vials were kept under different photoperiods as reared parasitoids, which had four levels: 0L:24D, 8L:16D, 16L:8D, and 24L:0D, and under a light intensity of 3000 lx [31].

### 2.3. Examining the Behavior of Female Parasitoids

We defined the pre-oviposition period as the interval between host inoculation and the first egg laid, as the parasitoids are synovigenic, meaning that oogenesis occurs after females are stimulated by feeding on the host hemolymph [32].

The developmental stages of the parasitoid offspring were defined as follows: the time elapsed between the first egg laid and the first larva to emerge was the egg stage; the time between the emergence of the first larva and the first cocoon was the larval stage; the pupal stage was the time between the first cocoon and the first adult male or female to emerge; and the egg–pupal stage was the time between the first egg laid and the first adult male or female to emerge. Each parasitized host was examined under a microscope twice a day.

A host was counted as successfully parasitized when the parasitoid deposited its eggs on it. If no eggs were observed on the host, it was recorded as a parasitism failure. Moreover, the parasitism success rate refers to the proportion of those eggs that successfully completed development and emerged as adults. The parasitism rate for each parasitoid species was calculated as the percentage success of the 35 replicates. When the parasitoids eclosed from the rearing vial, their development was thought to have been effectively completed.

### 2.4. Brood Size and Male Ratio of Parasitoid Offspring

The total number of male and female parasitoid progeny was counted. The female parasitoids were identified by their obvious ovipositor [33]. The male parasitoid ratio at emergence was the percentage of male offspring (the number of males: the total number of offspring).

### 2.5. Statistical Analyses

All statistical analyses were conducted using SPSS version 22.0. Parasitism and emergence rates, treated as binary variables, were analyzed using Chi-square tests with a significance level of α < 0.05 after verifying that all test assumptions were met. Variables including pre-oviposition time, egg stage duration, larval stage duration, pupal stage duration, egg-to-pupal developmental period, brood size, and male ratio were analyzed by one-way ANOVA followed by LSD post hoc tests at α < 0.05. Prior to analysis, the normality of these data was assessed, and for datasets that violated normality assumptions, arcsine square root transformation was applied before conducting the ANOVA.

## 3. Results

### 3.1. Parasitism Rate and Offspring Emergence Rate of Female S. sichuanensis Under Different Photoperiods

The parasitism rate of female *S. sichuanensis* on *T. gebleri* larvae differed significantly under different photoperiods (χ^2^ = 81.997, df = 4, *p* < 0.05), whereas the offspring emergence rate showed no significant differences (χ^2^ = 10.965, df = 4, *p* > 0.05) (Table 1). In complete darkness, the parasitism rate was significantly lower, with approximately one-quarter of the females failing to successfully parasitize the larvae. In contrast, under the other three photoperiod conditions, the parasitism rate reached 100%, and the offspring emergence rate exceeded 85% in all cases (Table 1).

### 3.2. Pre-Oviposition Period of Female S. sichuanensis Under Different Photoperiods

**Figure 1:**
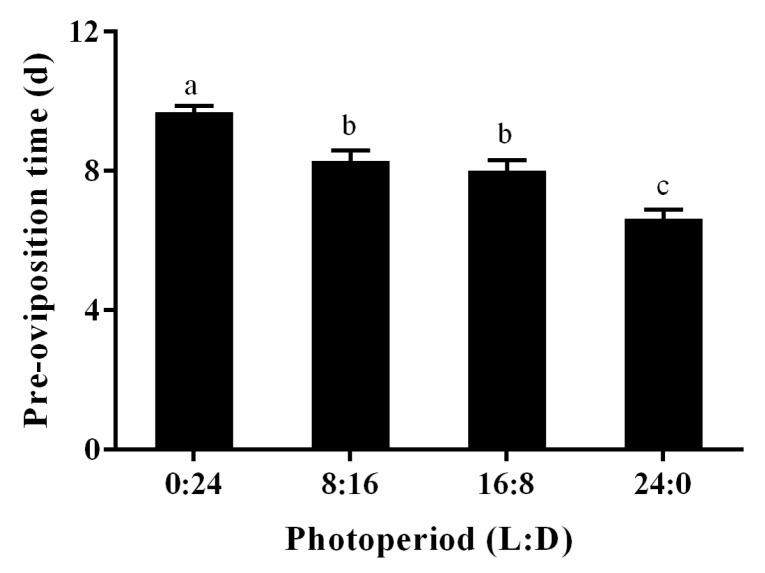
The pre-oviposition time of *S. sichuanensis* females under different photoperiods. Data are means ± SE of thirty-five replicates. Different letters over the columns indicate a significant difference among different groups using ANOVA performed on LSD (*p* < 0.05).

Under different photoperiods, the pre-oviposition period of female *S. sichuanensis* significantly decreased with increasing light duration (F = 18.618, df = 3130, *p* < 0.05). Under complete darkness, the pre-oviposition period was the longest, averaging 9.7 days. Under photoperiods of 8L:16D and 16L:8D, the differences were minimal, with an average of approximately eight days. Under constant light, the pre-oviposition period was shortened by approximately 3 days compared with complete darkness (Figure 1).

### 3.3. Egg Stage, Larval Stage, Pupal Stage, and Egg-to-Pupa Development Period of S. sichuanensis Offspring Under Different Photoperiods

**Figure 2:**
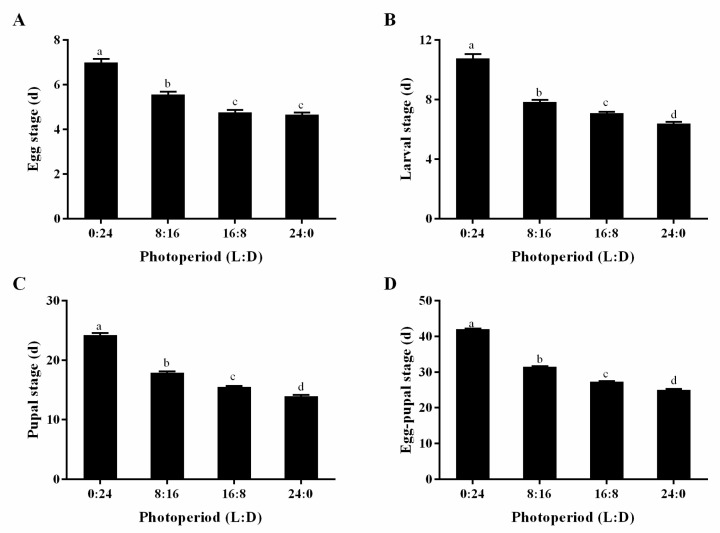
The development stage of *S. sichuanensis* under different photoperiods. (**A**): egg stage, (**B**): larval stage, (**C**): pupal stage, (**D**): egg–pupal stage. Data are means ± SE of thirty-five replicates. Different letters over the points indicate a significant difference among different groups using ANOVA performed on LSD (*p* < 0.05).

Under different photoperiods, the egg stage duration of *S. sichuanensis* offspring significantly decreased with increasing light duration (F = 75.302, df = 3130, *p* = 0.0001). In complete darkness, the egg stage was the longest, averaging seven days. Under the 8L:16D photoperiod, it was significantly shortened to an average of 5.6 days. With photoperiods of 16L:8D and 24L:0D, it further decreased to less than 5 days (Figure 2A).

Under different photoperiods, the larval stage duration of *S. sichuanensis* offspring significantly decreased as light duration increased (F = 125.776, df = 3121, *p* = 0.0001). In complete darkness, the larval stage was the longest, averaging 10.8 days. Under an 8L:16D photoperiod, it shortened significantly by approximately 3 days, averaging 7.8 days. With a 16L:8D photoperiod, the duration further decreased to an average of 7.1 days, and under constant light (24L:0D), it was reduced to an average of 6.4 days (Figure 2B).

Under different photoperiods, the duration of the pupal stage of *S. sichuanensis* offspring decreased significantly with increasing light duration (F = 317.893, df = 3121, *p* = 0.0001). In complete darkness (0L:24D), the pupal stage was the longest, averaging 24.2 days. Under an 8L:16D photoperiod, it was significantly shortened by approximately 6 days, averaging 17.9 days. With a 16L:8D photoperiod, the duration further decreased to an average of 15.5 days. Under constant light (24L:0D), the pupal stage was reduced to an average of 13.9 days, which was 10 days shorter than that under complete darkness (Figure 2C).

The egg-to-pupal developmental period of *S. sichuanensis* offspring varied significantly under different photoperiods (F = 1019.003, df = 3121, *p* = 0.0001). Under complete darkness (0L:24D), the egg-to-pupa period was the longest, averaging 42 days. With an 8L:16D photoperiod, it was significantly shortened by approximately 10 days, averaging 31.4 days. Under the 16L:8D photoperiod, the duration decreased further to an average of 27.3 days. Under constant light (24L:0D), the egg-to-pupa period was the shortest, averaging 25 days, which was 17 days shorter than that under complete darkness (Figure 2D).

### 3.4. Offspring Characteristics of S. sichuanensis Under Four Photoperiod Treatments

In the three treatments with dark phases, the number of male offspring ranged between 2 and 3 males per tube. Under constant light, the number of male offspring significantly decreased to an average of 1.7 males per tube, approximately 1.3 fewer than the maximum value (Table 2).

The number of female offspring was consistently higher than the number of male offspring across all treatments. In the three dark phase treatments, the number of female offspring ranged from 50 to 55 females per tube, with minimal differences. Under constant light, the number of female offspring significantly decreased, with approximately 18 fewer females per tube than the maximum value (Table 2).

The total number of offspring followed a pattern similar to that of the female offspring. In the three treatments with dark phases, the total offspring count ranged from 53 to 57per tube, with minor differences. Under constant light, the total number of offspring significantly decreased to approximately 19 fewer offspring per tube than the maximum value (Table 2). The proportion of male offspring was not significantly different across the four treatments, ranging between 4.3 and 5.2%, with a maximum and minimum difference of less than 1% (Table 2).

## 4. Discussion

Most insects have evolved seasonal biological clocks that enable them to measure daily photoperiods and use this information as a cue to coordinate their development and physiology [21]. Studies have shown that the photoperiod significantly affects the egg developmental duration of *S. sichuanensis*. Under complete darkness, the egg duration was the longest, averaging seven days. Under an 8L:16D photoperiod, it was significantly shortened to an average of 5.6 days, and under constant light conditions, it was the shortest, averaging less than five days. This is consistent with the findings of Chu et al. in *A. ypsilon*, where egg duration decreased with increased light exposure [29]. Generally, insect development slows in darkness but accelerates under light conditions, indicating that the photoperiod directly affects developmental rates. The larval developmental duration of *S. sichuanensis* was similarly affected by the photoperiod and decreased with longer light durations. Under complete darkness, the larval duration was the longest, averaging 10.8 days. Under the 8L:16D photoperiod, it was shortened by approximately three days. Under 16L:8D, the duration further decreased to an average of seven days, and at 24L:0D, it was the shortest, reduced by approximately four days. This aligns with the findings of Zhang et al. on *Encarsia formosa*, who reported similar patterns [4].

Photoperiodic information is perceived by specific photoreceptors and transmitted to a photoperiodic clock, which measures the length of the photophase or scotophase to determine whether the photoperiod is a long day (LD) or short day (SD) [3]. LD or SD information is then conveyed to the regulatory system, which induces diapause/reduced growth rate or non-diapause/faster growth rate responses. Evidence suggests that the circadian clock is involved with the photoperiodic clock [5,6,34].

The photoperiod significantly affects the development of insect reproductive organs, influencing oviposition and reproductive efficiency across different species [35]. Research has shown that under constant light conditions, *S. sichuanensis* produced the lowest number of male and female offspring. The average number of male offspring was approximately 1.3 fewer per tube than the maximum observed number, whereas the number of female offspring per tube was reduced by approximately 18. Similarly, the total number of offspring per tube was reduced by approximately 19. The highest number of female offspring and total progeny was observed under the 8L:16D photoperiod, followed by the 16L:8D photoperiod. Under complete darkness, female offspring and total progeny were reduced by approximately three per tube compared with the maximum.

This pattern aligns with the findings for *A. ypsilon*, where oviposition was the lowest under constant light, moderate under complete darkness, and highest at 12L:12D [29]. Similarly, *T. dendrolimi* exhibits maximum oviposition under 15L:9D and minimum oviposition under 3L:21D photoperiods [17]. These observations emphasize the critical role of light in insect reproduction, with both excessive and insufficient light being detrimental.

The photoperiod influences insect reproduction by altering the levels of JH and ecdysteroids. JH promotes vitellogenesis and egg maturation, thereby enhancing reproduction [35]. It also regulates extracellular matrix gene expression, facilitates ovulation, and maintains egg shape [36]. Ecdysteroids play key roles in oogenesis, including the regulation of yolk protein synthesis in the fat body and the influence on egg maturation and oviposition [37,38]. Moreover, there is evidence of an interaction between JH and ecdysteroids in the regulation of insect reproduction [39,40].

The pre-oviposition period of *S. sichuanensis* is significantly shortened with increasing light duration. Under complete darkness, the average pre-oviposition period was the longest, at 9.7 days. Under constant light, it was the shortest, approximately 3 days shorter than in complete darkness. The influence of photoperiod on the pre-oviposition period varies by species. For example, under short photoperiods, *Mythimna separata* has significantly longer pre-oviposition periods than under long photoperiods, similar to *S. sichuanensis* [41]. Conversely, constant light extends the pre-reproductive period in *H. armigera* [28]. The photoperiod had no significant effect on the pre-oviposition period of *Chrysoperla sinica* during diapause termination [42].

The photoperiod also affected the parasitism rate of *S. sichuanensis*. Under complete darkness, the parasitism rate of *M. subtruncatus* larvae was the lowest at only 74.3%. Under 8L:16D, 16L:8D, and constant light conditions, the parasitism rates reached 100%. Similar patterns were observed in other species. For example, *Anagyrus pseudococci* has the highest parasitism rate under a 12L:12D photoperiod, with both long and short photoperiods being unfavorable [43]. Extended photoperiods increase the lifespan of *Bracon hebetor*, enhancing parasitism rates [20]. These results indicated that appropriate photoperiods could enhance the efficiency of parasitism and improve biological control.

Species of the genus *Sclerodermus*, such as *S. guani* and *S. sichuanensis*, are widely used as biological control agents in China [1]. They target pests such as longhorn beetles, jewel beetles, and weevils that bore tree bark or trunks. After parasitizing these pests, *Sclerodermus* offspring develop within the larval tunnels under low-light conditions. It was hypothesized that light conditions might have minimal effects on *Sclerodermus* development and reproduction. However, research has indicated that the photoperiod plays a significant role [12,13]. In China, long summer days combined with suitable temperatures form a biological clock that accelerates *Sclerodermus* development, even under constant temperature conditions. The experimental results suggest that the optimal photoperiod conditions for rearing *S. sichuanensis* are 8L:16D, which maximizes parasitism rates, shortens pre-oviposition periods, reduces developmental duration, and increases the total number of offspring. However, similar to many insects, excessive light exposure is detrimental to reproduction in *S. sichuanensis.*

## Figures and Tables

**Table 1:** The parasitism ability of *S. sichuanensis* under different photoperiods.

Photoperiod (L:D)	Replicates (n)	Parasitism (n)	Parasitism Rate (%)	Successful Parasitism Number (n)	Successful Parasitism Rate (%)
0:24	35	26	74.3 b	25	96.2 a
8:16	35	35	100.00 a	33	94.3 a
16:8	35	35	100.00 a	33	94.3 a
24:0	35	35	100.00 a	31	88.6 a
χ^2^	−	−	81.997	−	5.127
*p*	−	−	*p* < 0.05	−	*p* > 0.05

Note: Data are means ± SE of thirty-five replicates. Values with different lowercase letters in the same column are significantly different at the 0.05 level.

**Table 2:** The brood size and male ratio of *S. sichuanensis* progeny.

Photoperiod (L:D)	Number of Male Offspring (n)	Number of Female Offspring (n)	Total Number of Offspring (n)	Male Ratio (%)
0:24	2.4 ± 0.3 ab	50.8 ± 3.5 a	53.1 ± 3.6 a	4.8 ± 0.5 a
8:16	2.3 ± 0.2 ab	53.7 ± 3.1 a	56.1 ± 3.8 a	4.3 ± 0.4 a
16:8	3.0 ± 0.3 a	53.1 ± 3.6 a	55.9 ± 3.2 a	5.2 ± 0.4 a
24:0	1.7 ± 0.3 b	35.5 ± 3.1 b	37.3 ± 3.3 b	5.1 ± 0.4 a
df	3121	3121	3121	3121
F	4.069	6.76	6.808	0.906
*p*	0.0086	0.0003	0.0003	0.4405

Note: Data are means ± SE of thirty-five replicates. Values with different lowercase letters in the same column are significantly different at the 0.05 level.

## Data Availability

The original contributions presented in this study are included in the article. Further inquiries can be directed to the corresponding author.

## Abbreviations

The following abbreviations are used in this manuscript:

LD	Long day
SD	Short day
JH	Juvenile hormone
Kr-h1	Krüppel homolog 1
JHAMT	JH acid O-methyltransferase
CA	corpora allata
20E	20-Hydroxyecdysone
ANOVA	Analysis of variance
LSD	Least significant difference
